# Interrater Reliability for Classifying Craniofacial Microsomia Severity: A Call for Objective Evaluation

**DOI:** 10.1177/10556656231216557

**Published:** 2023-11-22

**Authors:** Elsa M. Ronde, Jitske W. Nolte, Alfred G. Becking, Corstiaan C. Breugem

**Affiliations:** 1 Department of Plastic, Reconstructive and Hand Surgery, 26066 Amsterdam UMC, 522567 University of Amsterdam, Amsterdam, the Netherlands; 2 Department of Oral and Maxillofacial Surgery, 26066 Amsterdam UMC, University of Amsterdam, Amsterdam, the Netherlands; 3 Amsterdam Reproduction and Development research institute, Amsterdam, the Netherlands; 4 Amsterdam UMC Expert Center for Cleft, Craniofacial and Airway Disorders, Amsterdam, the Netherlands

**Keywords:** hemifacial microsomia, severity classification, facial morphology, imaging, OMENS, PAT-CFM‌, reliability‌

## Abstract

**Objective:**

The severity of craniofacial microsomia (CFM) is generally classified using the Orbit, Mandible, Ear, Soft tissue, Nerve (OMENS) classification score. The global assessment of the Phenotypic Assessment Tool for Craniofacial Microsomia (PAT-CFM), is a pictorial modification of the OMENS classification. The aim of this study was to assess the interrater reliability of the PAT-CFM global assessment score.

**Design:**

In this prospective cohort study, three clinicians completed the global assessment form of the PAT-CFM. The mandible was classified based on orthopantomogram- and/or computed tomography images.

**Participants:**

Consecutive patients with CFM or microtia.

**Main Outcome Measure:**

Interrater agreement was calculated using the weighted Krippendorff alpha (α), with 95% confidence intervals (CI).

**Results:**

In total, 53 patients were included (106 hemifaces). The reliabilities of the main classification components ranged from high for the mandible (α = 0.904 [95% CI 0.860–0.948]) and ear (α = 0.958 [95% CI 0.934–0.983]) subscales, to tentative for the orbital summary score (α = 0.682 [0.542–0.821]), and nerve summary score (α = 0.782 [0.666–0.900]) subscales.

**Conclusions:**

The reliability of the ear and radiographic mandible scales of the PAT-CFM global classification were high, while the orbit, facial nerve and soft tissue subscales may have limited reliability. Research focusing on radiographic severity scores for hypoplasia of the orbits and soft tissues, as well as objective measures for overall facial hypoplasia using non-ionizing forms of imaging for early classification, are warranted.

## Introduction

Craniofacial microsomia (CFM) is a rare condition with an estimated global prevalence between 1:5500 and 1: 26 000 births.^1-3^ Although there is no clear consensus on the diagnostic criteria of CFM,^4,5^ it is generally characterized by a varying degree of hypoplasia of the mandible, ears, facial soft tissues and/or facial nerves with or without other craniofacial anomalies such as ear tags and epibulbar dermoids.^6^ Minimal diagnostic criteria for CFM include mandibular hypoplasia,^7-9^ microtia,^10-13^ or a combination of two or more anomalies associated with CFM.^3,14-16^

The most commonly used classification tool for CFM is the Orbit, Mandible, Ear, Nerve and Soft tissue (OMENS) classification tool,^4^ which was developed by Vento et al. to classify the degree of hypoplasia or decreased function in the aforementioned structures.^17^ The OMENS was modified into a pictorial version by Gougoutas et al.,^18^ which was in turn further adapted by Birgfeld et al. into the Phenotypic Assessment Tool for Craniofacial Microsomia (PAT-CFM).^19^ The PAT-CFM consists of global, detailed and radiographic assessments, corresponding essentially to the modified pictorial OMENS, assessment of craniofacial anomalies, and radiographic assessment of the orbits and mandible, respectively. The radiographic assessment of the mandible, as in the original OMENS, corresponds to the classification developed by Pruzansky,^7^ and modified by Kaban et al. (Pruzansky-Kaban classification).^9,20^ The PAT-CFM score is also included in the International Consortium for Health Outcome Measurement (ICHOM) standard set for CFM,^21^ which is recommended for use by the European Reference Network guideline for CFM for all member centers.^16^

Previous studies have compared in-person and photographic assessment of the PAT-CFM, as well as photographic assessment using two- and three-dimensional photography.^22,23^ However, these assessments have been performed by largely the same group of researchers, who were also involved in developing the PAT-CFM, and the primary intent of these analyses was not to evaluate the interrater reliability of a single evaluation method. The aim of the current study was to assess the interrater reliability of the global assessment tool of the PAT-CFM.

## Methods

The Medical Ethics Review Committee of the Academic Medical Center exempted this study from full review (W21_319 # 21.354). The Guidelines for Reporting Reliability and Agreement Studies (GRRAS) checklist were followed (Supplementary file 1).^24^

### Patients

Consecutive patients who visited the multidisciplinary outpatient clinic for craniofacial microsomia and microtia at the Amsterdam University Medical Centers, location Academic Medical Center from January 2021 until February 2023 were assessed. Patients were eligible for inclusion if they had unilateral or bilateral microtia, or craniofacial microsomia as defined by the ICHOM criteria.^21^ Patients with seemingly isolated microtia were also included, as isolated microtia is often considered (one of the) minimal criteria for craniofacial microsomia.^6^ Patients with diagnosed syndromes such as CHARGE or auriculocondylar syndrome were excluded.

### Interpretation of Classification Components

The global assessment scale of the PAT-CFM^19,21^ for classification, which included assessment of the orbits, mandible, ears, facial nerves, soft tissues and lateral clefting (Table 1) was used. For clarity, non-radiographic assessment is referred to as clinical assessment. Orbital size and displacement were assessed clinically. The mandible was evaluated using the radiographic assessment scale (Pruzansky-Kaban) and computed tomography (CT) or orthopantomogram (OPG) images, if available. The clinical mandible and soft tissue scores were combined under the ‘soft tissue’ heading, considering that the mandible and soft tissue scores have been reported as a combined score in the past,^22^ and that distinguishing between skeletal and soft tissue hypoplasia without radiographic imaging is challenging.^25^ Facial nerve function was assessed by requesting the patient to lift their eyebrows, close their eyes, as well as smile and purse their lips with maximal effort. Any observed facial nerve weakness was classified as abnormal function. The orbit and nerve subscales were summarized as per the original OMENS.^17^ See Table 1 for a full overview of the classification components.

**Table 1.** Classification Components.

Component	Value	Definition
Orbital size (OS)	OS0	Normal size
	OS1	Abnormal size
Orbital displacement (OD)	OD0	Normal position
	OD1	Inferior displacement
	OD2	Superior displacement
Orbital summary score (O)	O1	Abnormal size, normal position
	O2	Normal size, abnormal position
	O3	Abnormal size and position
Mandible (M)^a^	M0	Normal
	M1	Small mandible and short ramus
	M2A	Abnormally shaped and short ramus
	M2B	Abnormally shaped and short ramus and anterior/inferior/medial displacement of the glenoid fossa and small condyle.
	M3	Absence of glenoid fossa (no TMJ)
Ear (E)	E0	Normal
	E1	All parts present, mild deformity.
	E2	Auricle ½ - ⅔ smaller and parts absent.
	E3	Severely malformed, often peanut shaped.
	E4	Anotia
Nerve: brow (NB)	NB0/NO0/NS0/NL0	Normal function
Nerve: orbit (NO)	NB1/NO1/NS1/NL1	Palsy
Nerve: smile (NS)		
Nerve: lower lip (NL)		
Nerve summary score (N)	N0	No facial nerve involvement
	N1	Upper facial nerve involvement (temporal and zygomatic branches)
	N2	Lower facial nerve involvement (buccal, mandibular and cervical branches)
	N3	All branches affected
Soft tissue^b^	S0	No hypoplasia
	S1	Minimal hypoplasia
	S2	Moderate hypoplasia
	S3	Severe hypoplasia
Lateral cleft	LC0	No cleft
	LC1	Cleft terminates medial to anterior border of masseter
	LC2	Cleft terminates lateral to anterior border of masseter

^a^ Radiographically assessed using computed tomography and/or orthopantomogram images, if available.

^b^ Combined clinical mandible and soft tissue scores.

*Adapted from:*

*Vento AR, Labrie RA, Mulliken JB. The O.M.E.N.S. Classification of Hemifacial Microsomia. Cleft Palate Craniofac J. 1991;28(1):68-77;*

*Birgfeld CB, Luquetti DV, Gougoutas AJ,* et al. *A phenotypic assessment tool for craniofacial microsomia. Plast Reconstr Surg. 2011;127(1):313-20;*

*Birgfeld CB, Heike CL, Saltzman BS,* et al. *Reliable classification of facial phenotypic variation in craniofacial microsomia: a comparison of physical exam and photographs. Head Face Med. 2016;12:14*

### Assessment Procedure

Three clinicians (a plastic surgeon with 15 years of experience, a maxillofacial surgeon with 10 years of experience and a junior doctor with six months of prior experience) assessed patients independently during a single regular in-person outpatient clinic visit. Prior to the start of inclusions, the maxillofacial surgeon briefed the plastic surgeon and junior doctor on assessing the Pruzansky-Kaban score, as well as interpreting CT/OPG images. The assessment procedure was piloted in two patients. Patient characteristics were collected from the outpatient visit note in the electronic medical file.

### Statistical Analyses

Data were analyzed using SPSS version 28.0.1.1 and R version 4.2.1, via the RStudio interface. Patient characteristic data are presented as number (%) and median (interquartile range [IQR]), depending on the type of data. Interrater reliability was measured using the Krippendorff alpha (α), due to its applicability for ordinal and missing data.^26^ The irrCAC package version 1.0 was used to calculate KA with ordinal weighting. For the orbital displacement, orbit summary score and nerve summary score, custom weights matrices were defined based on the ordinal weights, to reflect equal rank order within these scales (e.g., in the orbital displacement category “superior displacement” and “inferior displacement” are both one rank order removed from “normal position”, and two rank orders removed from each other, even though the category values are 2, 1 and 0, respectively [see Table 1 for category values]). If assessment of the original anomaly wasn’t possible due to reconstruction, then the assessment was considered missing. We calculated 95% confidence intervals using the same package. Reliability was classified as high if α ≥ 0.800, tentative if 0.800 < α ≥ 0.667, and low if α < 0.667.^27^

## Results

In total, 53 patients were assessed (106 hemifaces), where 47 patients had CFM according to the ICHOM criteria, and 6 had isolated microtia. Five patients (9%) had bilateral involvement. The median age of patients at assessment was 11 (IQR 5–15) years, and 27 (51%) were male. See Table 2 for an overview of patient characteristics.

**Table 2.** Patient Characteristics (n = 53).

Characteristic	N (%) All patients, n = 53	N (%) M/A only, n = 6	N (%) CFM^a^, n = 47
Age, years	Median 11 (IQR 5–15)	Median 11 (IQR 3–13)	Median 9 (6–15)
Sex, male	27 (51)	4	23 (51)
Laterality			
Unilateral, right	30 (57)	3	27 (57)
Unilateral, left	18 (34)	3	15 (32)
Bilateral	5 (9)	0	5 (11)
OMENS score^b^			
*Orbit*			
O0	39 (74)	6	33 (70)
O1	5 (9)	0	5 (11)
O2	4 (8)	0	4 (9)
O3	5 (9)	0	5 (11)
*Mandible*			
M0	7 (13)	3	4 (9)
M1	15 (28)	0	15 (32)
M2A	5 (9)	0	5 (11)
M2B	10 (19)	0	10 (21)
M3	2 (4)	0	2 (4)
Unable to assess^c^	14 (26)	3	11 (23)
*Ear*			
E0	3 (6)	0	3 (6)
E1	5 (9)	0	6 (13)
E2	8 (15)	2	6 (13)
E3	30 (57)	4	26 (55)
E4	2 (3)	0	2 (4)
Reconstructed^d^	5 (9)	0	5 (11)
*Nerve*			
N0	28 (53)	6	22 (47)
N1	5 (9)	0	5 (11)
N2	7 (13)	0	7 (15)
N3	13 (25)	0	13 (28)
*Soft tissue*			
S0	19 (36)	6	13 (28)
S1	18 (34)	0	18 (38)
S2	12 (23)	0	12 (26)
S3	4 (8)	0	4 (9)

M/A: microtia/anotia; CFM: craniofacial microsomia; ICHOM: International Consortium of Health Outcome Measurement; OMENS: Orbit, Mandible, Ear, Nerve, Soft tissue.

^a^ According to ICHOM criteria.

^b^ For bilaterally affected patients, the more severely affected hemiface is included.

^c^ No radiographic imaging.

^d^ Preoperative classification unknown.

All patients were evaluated by at least two clinicians. Three ratings were available for 43 patients and two for 10 patients due to absences. We found high reliabilities for the mandible (α = 0.904 [95% CI 0.860–0.948]) and ear (α = 0.958 [95% CI 0.934–0.983]) subscales. The reliability of the orbit summary score (α = 0.682 [0.542–0.821]), nerve summary score (α = 0.782 [0.666–0.900]) and subscales for soft tissue (α = 0.760 [0.667–0.853]), as well as facial nerve function of the brow (α = 0.750 [0.609–0.892]) and orbit (α = 0.715 [0.508–0.922]), were tentative. Reliabilities for subscales on orbital size (α = 0.645 [0.454–0.837]), orbital displacement (α = 0.664 [0.472–0.857], as well as facial nerve function of the upper (α = 0.566 [0.335–0.797]) and lower lip (α = 0.642 [0.492–0.791]) were low. See Table 3 for an overview of these results.

**Table 3.** Interrater Agreement Results (n = 106).

Classification component	Krippendorff α^a^ (95% CI)
Orbital size	0.645 (0.454–0.837)
Orbital displacement	0.664 (0.472–0.857)
Orbit summary score	0.682 (0.542–0.821)
Mandible	0.904 (0.860–0.948)
Ear	0.958 (0.934–0.983)
Nerve: brow	0.750 (0.609–0.892)
Nerve: orbit	0.715 (0.508–0.922)
Nerve: smile	0.566 (0.335–0.797)
Nerve: lip	0.642 (0.492–0.791)
Nerve summary score	0.782 (0.666–0.900)
Soft tissue^b^	0.760 (0.667–0.853)
Macrostomia	0.737 (0.482–0.992)

^a^ High reliability: α ≥ 0.800, tentative reliability: 0.800 < α ≥ 0.667, low reliability: α < 0.667.

^b^ Soft tissue and non-radiographic mandible score.

A pairwise comparison of raters suggested similar agreement among raters, where agreement between the maxillofacial surgeon and the junior doctor was highest (Supplementary file 2).

## Discussion

In this study, we assessed the reliability of the global classification of the pictorial modification of the OMENS classification tool at the multidisciplinary outpatient clinic for craniofacial microsomia and microtia at a national referral center in the Netherlands. The results of the current study suggest a high reliability of the ear and radiographic mandible (Pruzansky-Kaban) subscales, and a possibly limited reliability for the orbit, facial nerve and soft tissue subscales.

The higher reliabilities of the ear and Pruzansky-Kaban subscales may be explained by the anatomic descriptions included in these classification components. For the ear scale, severity is essentially based on the presence or absence of auricular structures, while the Pruzansky-Kaban relies on assessment of the size and morphology of the mandible, condyle, glenoid fossa and temporomandibular joint. The ear scale, which essentially corresponds to the Marx classification,^28^ seems suitable for reliable classification of the severity of the ear malformation, especially considering that it can be applied to even the youngest of patients. Adding specific descriptions of minimal anatomic requirements that correspond to surgical classifications^29^ (ie, a [possibly malformed] concha or concha-type microtia for E2, and a cartilaginous remnant and lobule or lobule-type microtia for E3), as well as a separate category for atypical cases could improve this scale further. Higher correlation and reliability scores were also found for the ear subscale by two studies by Birgfeld et al. comparing in-person and photographic assessment, as well as assessments made using two-dimensional or three-dimensional images.^22,23^ To our knowledge, previous studies have not assessed reliability of in-person assessments specifically, and neither of these studies evaluated the radiographic assessment of the mandible.

Conversely, the orbit and soft tissue scales only contain global descriptors (eg, “inferior displacement” and “moderate hypoplasia”) which are more open to interpretation and require a reference point for what can be considered “normal”. In both previous studies assessing the reliability of the OMENS/PAT-CFM scale, the reliability of the orbit scale has been among the lowest.^22,23^ The clinicians noted several aspects that made assessing the orbit subscales challenging, in particular. First, it was difficult to visually identify a true horizontal plane in patients with orbital asymmetry to distinguish abnormalities in position and size, especially in cases of concurrent asymmetry in the lower half of the face. Varying head positions, as well as any asymmetries of the eyelids also made these parameters more challenging to assess. Although the PAT-CFM includes a radiographic assessment of the orbits, it is similar to the clinical orbit score, and it doesn’t include any quantifiable anatomic parameters.^19^ To our knowledge, no studies have reported on this radiographic scale, possibly due to its unclear clinical relevance. Previous studies have noted morphological changes with increasing severity of both the maxilla^30^ and zygoma,^31^ but the clinical relevance of these changes remains unclear. It may be worth considering whether it is necessary to distinguish between orbital size and displacement when globally assessing and classifying orbital asymmetry, especially considering the possibly tentative reliability of doing so. Instead, clinicians could consider a simple dichotomous assessment of orbital asymmetry (ie, present/absent) for global assessment. Similarly, the current clinical relevance of the soft tissue scale may be called into question. Next to containing only global descriptors, the soft tissue scale is very similar to the clinical mandible scale, considering that distinguishing between Pruzansky-Kaban 2A and 2B mandibular hypoplasia without radiographic imaging is not currently feasible. Previous studies have identified involvement of the masseter, pterygoid and temporal muscles,^32-34^ however, the current soft tissue scale does not mention tissue quality or muscle function. Future studies should focus on the clinical implications of orbital and soft tissue asymmetry, as well as the associated anatomical changes, in order to improve these scales in clinically relevant ways.

The limited reliability of the nerve subscales was not unexpected; even though clinicians agreed to consider any objectified weakness as “abnormal”, discriminating between completely normal function and slight weakness still presented challenges in practice. Possible weakness of the lower branches of the facial nerve was also difficult to distinguish from asymmetry due to occlusal canting, or mandibular and soft tissue hypoplasia in more severe cases. As discussed in previous studies, the facial nerve subscales lack a true gradation of severity.^25,35^ The interpretation of the nerve subscale also varies. Vento et al. discusses ‘facial nerve weakness’,^17^ while both Gougotas et al. and Birgfeld et al. use the term ‘paralysis’,^18,22^ which suggests different minimal severity requirements for considering facial nerve function abnormal. Although we did not formally assess palsy severity, most patients with abnormal nerve function in our cohort had some degree of weakness in motor function, while true paralysis was rare. Standardized assessment is needed for reliable and comparable results, and we recommend considering any weakness as abnormal function, and assessing the severity of this weakness separately using a validated scale. A systematic review from 2015 identified the Sunnybrook Facial Grading Scale as the current standard for reporting facial nerve function,^36^ and clinicians should consider this scale for classifying the facial nerve function in patients with craniofacial microsomia more comprehensively. Quantitative imaging-based static^37,38^ as well as dynamic^39^ assessments may be promising directions for future research.

Reliable severity classifications for facial hypoplasia are paramount for diagnostics, care and research, considering that higher degrees of facial hypoplasia have been associated with a higher occurrence of extracraniofacial anomalies,^25,40,41^ as well as feeding and breathing difficulties.^42,43^ Based on the current literature, most clinicians only use radiographic assessment for classifying the severity of mandibular hypoplasia.^35,44-47^ The lack of accepted radiographic criteria for all other assessment components makes the mandible the most important radiological assessment, but it is questionable whether the Pruzansky-Kaban classification can be used as a sole marker for facial hypoplasia. Several cohort studies have found correlations between (clinical) orbital, mandibular and soft tissue hypoplasia,^35,44-47^ but these findings are not consistent with radiographic studies assessing the relationship between mandibular involvement and orbital volume,^48^ temporal and zygomatic hypoplasia,^31^ or maxillary involvement.^30,49^ Similarly, a recent case series evaluating both soft tissue and bony hypoplasia using three-dimensional reconstructions of cone beam CT images, found significant correlations between bony and soft tissue hypoplasia in the malar and gonial regions, but not in the frontal, orbital or maxillary regions.^50^ This seems to indicate a possible discrepancy between clinical and radiographic assessments, and this may be due to the subjective nature of clinical assessments. Radiographic assessment is often delayed in routine clinical practice due to radiation concerns in very young patients. At our center, orthodontic records, including panoramic dental radiographic images, are routinely collected from 6 years of age, as recommended by the European Reference Network guideline for CFM.^16^ CT imaging is not routinely performed until an older age, unless indicated due to concurrent airway, feeding and/or hearing difficulties for diagnostics and subsequent surgical planning. Alternative objective evaluation methods are therefore needed to improve the reliability of early clinical assessments of facial hypoplasia, and these should be consistent with radiographic assessment in terms of severity. Three-dimensional surface imaging has been used to evaluate facial asymmetry in pediatric populations,^51-53^ and could be suitable for globally classifying facial hypoplasia in a quantitative and objective way without ionizing radiation. Future research should focus on implementing three-dimensional surface imaging for creating clinically relevant severity classifications.

This study has some limitations. First, the reliability of the full PAT-CFM scale was not evaluated to reduce the burden on both patients and clinicians, as this study was conducted during regular outpatient clinic visits. For this reason, assessments were also done simultaneously, which could have impacted clinicians’ evaluations. The impact of this was limited by not discussing assessments until after each clinician had filled out the form. Furthermore, our results saw wide confidence intervals in most classification components, possibly due to sample size limitations. Formal sample size requirements were not calculated prior to the study; instead, a target sample size of 50 patients (or 100 hemifaces) was set as a clinically feasible sample. According to sample size estimates by Krippendorff, a sample size of 95 would be sufficient for determining α for a scale with a maximum of five subcategories (such as the ear and mandible scales) at a 0.05 level of significance and a smallest acceptable α of 0.667.^54^ However, this estimate assumes equal distribution among subcategories, and this was not the case in our cohort. Therefore, true sample size requirements may have been higher.

All in all, this study identified several strengths and several limitations of using the PAT-CFM global classification scale routinely in clinical practice at a tertiary care hospital. We have outlined suggestions for improvement, although evaluating these suggestions was beyond the scope of the current study. As several of the global classification items and the suggestions for improvement may still be open to interpretation, a consensus meeting of experts of all involved specialties could be considered for evaluating current perspectives on the PAT-CFM global classification, as well as for suggesting implementation of a modified version in standard work-up.

## Conclusion

The PAT-CFM global classification can be reliably used to classify the severity of ear malformations and mandibular hypoplasia using radiographic assessment in patients with CFM or microtia. The orbit, facial nerve and soft tissue subscales may have limited reliability. Clinicians could consider using a dichotomous assessment for the presence of orbital asymmetry, and consider any facial nerve weakness as abnormal function, although any definitive changes to the PAT-CFM classification and its interpretation should be evaluated further, or discussed in a consensus meeting of experts of all specialties involved. Future research may focus on developing clinically relevant radiographic assessment severity scores for hypoplasia of the orbits and soft tissues, as well as objective measures for overall facial hypoplasia using non-ionizing forms of imaging, such as three-dimensional surface imaging.

## Supplemental Material

sj-docx-1-cpc-10.1177_10556656231216557 - Supplemental material for Interrater Reliability for Classifying Craniofacial Microsomia Severity: A Call for Objective Evaluation Supplemental material, sj-docx-1-cpc-10.1177_10556656231216557 for Interrater Reliability for Classifying Craniofacial Microsomia Severity: A Call for Objective Evaluation by Elsa M. Ronde, Jitske W. Nolte, Alfred G. Becking and Corstiaan C. Breugem in The Cleft Palate Craniofacial Journal

sj-docx-2-cpc-10.1177_10556656231216557 - Supplemental material for Interrater Reliability for Classifying Craniofacial Microsomia Severity: A Call for Objective Evaluation Supplemental material, sj-docx-2-cpc-10.1177_10556656231216557 for Interrater Reliability for Classifying Craniofacial Microsomia Severity: A Call for Objective Evaluation by Elsa M. Ronde, Jitske W. Nolte, Alfred G. Becking and Corstiaan C. Breugem in The Cleft Palate Craniofacial Journal
