# Supplementary material for: Interrater Reliability for Classifying Craniofacial Microsomia Severity: A Call for Objective Evaluation
Source: Cleft Palate Craniofac J. 2023 Nov 22;62(4):619–26. doi: 10.1177/10556656231216557 (PMC12075885; doi:10.1177/10556656231216557)
Supplement: sj-docx-1-cpc-10.1177_10556656231216557 - Supplemental material for Interrater Reliability for Classifying Craniofacial Microsomia Severity: A Call for Objective Evaluation [file sj-docx-1-cpc-10.1177_10556656231216557.docx]

Supplementary File 1. Guidelines for Reporting Reliability and Agreement Studies (GRRAS) checklist*.

| **No.** | **Item description** | **Reported on page** |
| --- | --- | --- |
| 1 | Identify in title or abstract that interrater/intrarater reliability or agreement was investigated. | 1 |
| 2 | Name and describe the diagnostic or measurement device of interest  explicitly. | 2, 3-4 |
| 3 | Specify the subject population of interest. | 3-4 |
| 4 | Specify the rater population of interest (if applicable) | n/a |
| 5 | Describe what is already known about reliability and agreement and provide a rationale for the study (if applicable) | 2 |
| 6 | Explain how the sample size was chosen. State the determined number of raters, subject/objects, and replicate observations. | 4  Sample size: 9-10 |
| 7 | Describe the sampling method. | 3 |
| 8 | Describe the measurement/rating process (e.g. time interval between repeated measurements, availability of clinical information, blinding). | 3-4 |
| 9 | State whether measurements/ratings were conducted independently. | 4 (9) |
| 10 | Describe the statistical analysis. | 4-5 |
| 11 | State the actual number of raters and subjects/objects which were included and the number of replicate observations which were conducted. | 5 |
| 12 | Describe the sample characteristics of raters and subjects (e.g. training, experience). | 4 |
| 13 | Report estimates of reliability and agreement including measures of statistical uncertainty. | 6, table 3 |
| 14 | Discuss the practical relevance of results. | 6-10 |
| 15 | Provide detailed results if possible (e.g. online) | Table 3 |

* Adapted from Kottner J, Audigé L, Brorson S et al. Guidelines for Reporting Reliability and Agreement Studies (GRRAS) were proposed. J Clin Epidemiol. 2011 Jan;64(1):96-106. doi: 10.1016/j.jclinepi.2010.03.002. Epub 2010 Jun 17. PMID: 21130355.
