# Supplementary material for: Interrater Reliability for Classifying Craniofacial Microsomia Severity: A Call for Objective Evaluation
Source: Cleft Palate Craniofac J. 2023 Nov 22;62(4):619–26. doi: 10.1177/10556656231216557 (PMC12075885; doi:10.1177/10556656231216557)
Supplement: sj-docx-2-cpc-10.1177_10556656231216557 - Supplemental material for Interrater Reliability for Classifying Craniofacial Microsomia Severity: A Call for Objective Evaluation [file sj-docx-2-cpc-10.1177_10556656231216557.docx]

**Supplementary table 1:** Interrater agreement results between raters (n=106)

| Classification component | Krippendorff α^a^  (95% CI) | | |
| --- | --- | --- | --- |
|  | Raters P and M | Raters P and J | Raters M and J |
| Orbital size | 0.593 (0.314-0.872) | 0.585 (0.320-0.851) | 0.700 (0.486-0.913) |
| Orbital displacement | 0.741 (0.518-0.962) | 0.554 (0.249-0.859) | 0.675 (0.426-0.924) |
| Orbit summary score | 0.747 (0.572-0.923) | 0.579 (0.364-0.793) | 0.702 (0.550-0.852) |
| Mandible | 0.868 (0.804-0.931) | 0.884 (0.812-0.956) | 0.954 (0.920-0.987) |
| Ear | 0.960 (0.929-0.991) | 0.953 (0.918-0.988) | 0.963 (0.934-0.993) |
| Nerve: brow | 0.606 (0.379-0.832) | 0.727 (0.516-0.939) | 0.867 (0.737-0.997) |
| Nerve: orbit | 0.756 (0.521-0.991) | 0.675 (0.401-0.948) | 0.755 (0.519-0.991) |
| Nerve: smile | 0.678 (0.407-0.949) | 0.543 (0.236-0.851) | 0.475 (0.175-0.774) |
| Nerve: lip | 0.669 (0.465-0.874) | 0.654 (0.442-0.865) | 0.592 (0.382-0.803) |
| Nerve summary score | 0.763 (0.608-0.919) | 0.837 (0.713-0.962) | 0.767 (0.609-0.924) |
| Soft tissue^b^ | 0.743 (0.626-0.859) | 0.717 (0.563-0.872) | 0.828 (0.743-0.913) |
| Macrostomia | 0.652 (0.276-1.000) | 0.883 (0.651-1.000) | 0.646 (0.317-0.976) |

P: plastic surgeon; M: Maxillofacial surgeon; J: junior doctor
a. High reliability: α ≥ 0.800, tentative reliability: 0.800 < α ≥ 0.667, low reliability: α < 0.667
b. Soft tissue and non-radiographic mandible score.

Red indicates that the score falls into a lower reliability category, green indicates that the score falls into a higher reliability category, and yellow indicates that the score falls into the same reliability category, compared to the ratings of three raters (table 3).
